# The effect of prostaglandin and gonadotrophins (GnRH and hCG) injection combined with the ram effect on progesterone concentrations and reproductive performance of Karakul ewes during the non‐breeding season

**DOI:** 10.1002/vms3.353

**Published:** 2020-09-20

**Authors:** Mohammad Ayaseh, Abdolah Mirzaei, Alidad Boostani, Mansur Mehrvarz

**Affiliations:** ^1^ Department of Clinical Sciences School of Veterinary Medicine Shiraz University Shiraz Iran; ^2^ Department of Animal Science Research Fars Agricultural and Natural Resources Research and Education Center Agricultural Research, Education and Extension Organization (AREEO) Shiraz Iran

**Keywords:** Ewes, GnRH, hCG, Non‐breeding season, Progesterone, Ram effect

## Abstract

The effect of prostaglandin and gonadotrophins (GnRH and hCG) combined with the ram effect on the progesterone (P4) concentrations and reproductive performance of Karakul ewes was investigated during non‐breeding season. Ewes (*n* = 93) received a male effect and were divided into two treatment groups including GnRH ‐ hCG (hCG, *n* = 32), GnRH ‐ GnRH (GnRH, *n* = 30) and a control (*n* = 31) group. This study was carried out from April (hormonal injection) to October (lambing). The first doses of GnRH (4.2 μg, Buserelin) were injected at the beginning of the study in treatment groups. These ewes were treated with hCG (250 IU) or the second GnRH dose five days later. All animals received two injections (ten days apart) of 150 μg PGF2α five days after the hCG or the second GnRH injection. Mating was initiated two days after the second prostaglandin injection and lasted for 34 days. Blood samples were collected by jugular venipuncture on days −10, −5, 0 (first PGF2α injection), 17 and 30 during the study. Pregnancy diagnosis was performed through transabdominal ultrasonography on day 40 after the removing of ram. Conception rate was 93.8, 90 and 87.1% in the hCG, GnRH and control groups, respectively. Lambing rate tended to increase in the hCG group compared with the control group (87.1 versus 58.1%; *p* < .1). There was no significant difference in P4 concentrations among studied groups in identical sampling times (*p* > .05). In conclusion, the administration of prostaglandin and hCG in combination with the ram effect tended to decrease lambing period. In other words this protocol tended to increase lambing rate at the first cycle. Treatment with hCG or GnRH did not increase serum P4 concentrations of treated Karakul ewes during the non‐breeding season.

## Graphical abstract

The administration of prostaglandin and hCG in combination with the ram effect may reduce lambing period and tended to increase lambing rate at the first cycle during the non‐breeding season. Administrations of double injection of GnRH not only had no effect on the progesterone concentrations but also couldn’t improve the reproductive performance of treated ewes. Treatment with hCG or GnRH on Day 5 after the GnRH administration did not increase serum P4 concentrations of treated Karakul ewes during the non‐breeding season

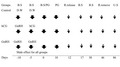

## 1 INTRODUCTION

Ewes are known as animals with seasonal reproductive activity. The anoestrous period covers the spring to early‐ or mid‐summer (Abecia, Forcada, & González Bulnes, 2012). The Karakul is a medium‐sized sheep, a fat‐tailed and multi‐purpose breed (source of milk, meat, tallow and fibre). The Karakul has an extended breeding season and the spring considered as anestrous season. Single lambs are the rule, though twins are not uncommon (Nsoso & Madimabe, 2003; Safdarian, Kafi, & Hashemi, 2006). Kafi, Safdarian, and Hashemi (2004) reported that the Karakul ram has the capability to be used for mating all year round. Several protocols have been developed to induce the reproductive function of ewes during the non‐breeding season (Abecia, Forcada, & González‐Bulnes, 2011; Ahmad Pampori, Ahmad Sheikh, Aarif, Hasin, & Ahmad Bhat, 2020; Rosa & Bryant, 2002, 2003).

The most commonly used synchronization methods are based on the use of progesterone or prostaglandin protcols. The intravaginal administration of progesterone with eCG injection is a common method of synchronization in sheep (Swelum, Alowaimer, & Abouheif, 2015). Although progesterone‐based programmes are preferred, these programmes are doubtful. There is a need to reconsider the protocols based on the use of progestagens for societal reasons such as animal health and welfare, food safety and the environmental impact (Gonzalez‐Bulnes, Menchaca, Martin, & Martinez‐Ros, 2020). Vinoles, Paganoni, Milton, Driancourt, and Martin (2011) reported that the adding antibiotics before the sponge insertion reduced the amount of mucus and odour compared with the control group. There was a possibility of the intravaginal sponges and antibiotic residues used to prevent vaginal infections (Berruga, Rodriguez, Rubio, Gallego, & Molina, 2008).

The use of prostaglandin F2α (PGF2α) reduced costs and are less environmental pollutants compared with progestagen intravaginal devices (Fierro, Gil, Vinoles, & Olivera‐Muzante, 2013). Prostaglandins are rapidly metabolized in the lung and have no tissue remnants (Davis, Fleet, Harrison, & Walker, 1980). Therefore, the use of PGF2α or its analogues is a good alternative synchronization method in sheep.

The use of gonadotropin releasing hormone (GnRH) or the male effect in PGF2α‐based protocols was reported previously (Mirzaei et al., 2017; Olivera‐Muzante, Gil, Fierro, Menchaca, & Rubianes, 2011). Human chorionic gonadotropin (hCG) and GnRH administrations on the day of mating or post‐mating were applied for improving the reproductive performance (conception, lambing, twining rate and litter size) of different breed (Akkaraman, fat tailed, Afshari × Booroola‐Merino crossbred, Anatolian Merino) ewes (Ahmadi & Mirzaei, 2016; Ataman, Aköz, Sarıbay, Erdem, & Bucak, 2013; Dursun, 2019; Rostami, Hajizadeh, Shahir, & Aliyari, 2017). A higher plasma P_4_ concentrations was reported in post‐mating hCG‐treated groups in Afshari × Booroola‐Merino crossbred ewes by Rostami et al. (2017). Injection of GnRH on the day of oestrus or at the time of mating and 7 or 9 days later increased serum P4 concentration.

and enhanced pregnancy rate and litter size (Hashem, El‐Azrak, Nour El‐Din, Taha, & Salem, 2015; Zonturlu et al., 2018). High pregnancy rates with natural mating were reported after treatment including GnRH injection at device insertion of progesterone during the out of breeding season (Martinez et al., 2015). In non‐breeding season, there was a tendency to a greater formation of CL and pregnancy rate in GnRH‐treated ewes compared with control (ram introduction alone) group (Jordan, Inskeep, & Knights, 2009). One injection of GnRH in a PGF2α‐based protocol combined with the ram effect enhanced lambing rate and litter size during non‐breeding season in Karakull ewes (Mirzaei et al., 2017).

The hypothesis being that GnRH or hCG treatments combined with the ram effect could induce ovulation in the ewes. The aim of the present study was to evaluate the effect of prostaglandin and GnRH or hCG in combination with the ram effect on progesterone concentrations and reproductive performance of Karakul ewes during the non‐breeding season. Plasma progesterone concentrations were used for assessing the response to hormonal injection, with regard to ovulation.

## 2 MATERIALS AND METHODS

### 2.1 Animals and flock management

The present study was performed in a Karakul flock during the non‐breeding (April to September) season in Saadat Shahr, Fars province, Iran. Saadat Shahr is located at latitude of 30° 3' N and longitude of 53° 7' E. Its altitude is 1892 m above sea level. A total of 93 non‐lactating Karakul fat‐tailed ewes were used during the study period.This study was carried out from April (hormonal injection) to October (lambing). The ewes were vaccinated and received anti‐parasitic drugs before starting the study. Ewes were divided into three groups, with respect to body condition score (BCS) and age, including two treatment groups (hCG and GnRH groups) and an untreated control group. Body condition score (BCS) of each ewe was determined through back vertebral palpation (1–5 points) (Pugh, 2002, 2003). The mean (± *SD*) of BCS of ewes in GnRH ‐ hCG (*n* = 32), GnRH ‐ GnRH (*n* = 30) and control (*n* = 31) groups were 3.20 ± 0.33, 3.28 ± 0.31 and 3.16 ± 0.27, respectively. The teeth formula and farm's records were the criteria for excluding very young and old ewes (Pugh, 2002, 2003). The mean (±*SD*) age of ewes in hCG, GnRH and control groups were 3.22 ± 0.38, 3.20 ± 0.36 and 3.18 ± 0.42 years old, respectively. They are multiparous (parity 2 and 3) and dry ewes. All ewes grazed on the natural pastures and were flushed with balance (alfalfa hay (23%), corn silage (67%) and barley grain (10%) ad libitum) from 3 weeks before breeding to the removing of ram (about 55 days). Mineral salt (containing 19.6% calcium and 9.6% phosphorus) and water were offered ad libitum. Ewes were kept away (3 months) from the rams before starting the study. At the time of first GnRH injection (Day –10 before the first injection of PGF2α), ewes and rams were kept in close proximity to each other through fences at nights.

### 2.2 Hormonal treatments

**FIGURE 1:**
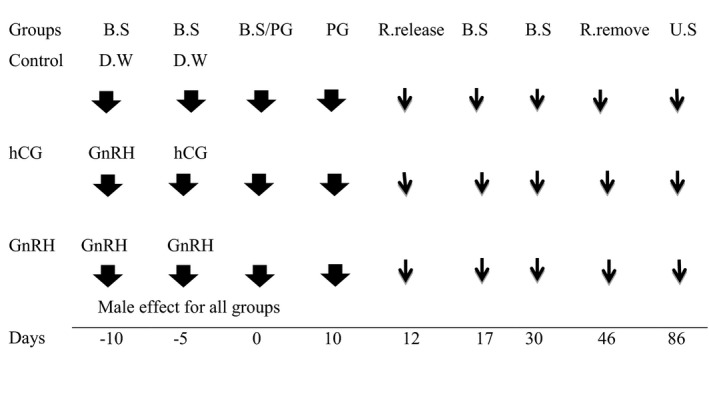
Scheme of treatment protocols in Karakul ewes during the experimental periods, relative to the first PGF2α injection (Day 0); B.S: blood sample; D.W: distilled water; PG: prostaglandin; R: ram; U.S: ultrasonography; Day 0: First dose of PGF2α injection

Ten days before the first injection of PGF2α (Day –10 before the first injection of PGF2α; Figure 1), ewes in the GnRH ‐ hCG (hCG) and GnRH ‐ GnRH (GnRH) groups received an intramuscular injection of GnRH (4.2 μg, Buserelin, 4.2 μg/ml, Aburaihan, Iran). This injection was given in order to induce ovulation or luteinize for mature and immature follicles, respectively. Five days later after the GnRH dose (Day –5 before the first injection of PGF2α; Figure 1), ewes were treated with hCG (250 IU, IM, LG Chem, Ltd.; South Korea; hCG group) or the second GnRH dose (4.2 μg, Buserelin; GnRH group) for inducing ovulation. Animals considered as a control group received distilled water as a placebo on the same days.

On day 0, ewes in all groups were given the first dose of PGF2α (150 μg, Cloprostenol, 75 μg/ml, Rooyan Darou, Iran). Ten days later, all ewes received a second PGF2α dose (150 μg, Cloprostenol). Mating was initiated two days after the second prostaglandin injection and lasted for 34 days later (from day 12 to 46 after the first injection of PGF2α, two oestrous cycles; Figure 1). Twelve fertile Karakul rams (four males per each treatment) with good body condition scores were released into the ewes. The hormonal treatments of the present study are shown in Figure 1

### 2.3 Sampling and Ultra‐sonographic examination

Blood samples (*n* = 93) were collected by jugular venipuncture on days −10, −5, 0, 17 and 30 during the study (Figure 1). Serums were separated through centrifugation at 1,500 × g for 10 min and were stored at −20°C. Serum P4 concentrations were determined using a validated commercial ELISA kit (IBL International, Hamburg, Germany). The intra‐assay and inter‐assay CV were 5.4% and 8.6%, respectively. The sensitivity of the assay was 0.1 ng/ml. The ewes with serum P4 concentrations of more than 1 ng/ml on day 0 (first dose of PGF2α injection) were considered as ovarian responders (ovulation) to hCG or second GnRH administration (Mirzaei et al., 2017). Pregnancy diagnosis was performed by transabdominal ultrasonography on days 40 after the removing of ram in standing position using a real‐time ultrasound scanner equipped with a 3.5 MHz convex probe (KX5200, Kaixin, China). The probe was applied at the inguinal region of right side after adding the gel (Aziz & Lazim, 2012). The conception rate (based on the pregnancy diagnosis), lambing rates, fetal mortality rate, litter size (lambs/lambed ewe) and weights of lambs were recorded for the evaluations. The reproductive performance of ewes was evaluated by calculating the following variables: Conception rate = (number of pregnant ewes at pregnancy diagnosis/number of exposed ewes) × 100; Lambing rate = (number of ewes lambing/number of exposed ewes) × 100; Fetal mortality rate = [(number of detected embryos at pregnancy diagnosis – number of lambs born)/number of detected embryos at pregnancy diagnosis] ×100. We recorded the data of lambing (lambing period) on two consecutive cycles. First and second cycles were considered from 151 to 159 and 166 to 181 days after ram release, respectively. Pregnant ewes conceived at induced and first spontaneous oestrus, they lambed during the first and second cycle, respectively. Overall lambing rate was defined as the proportion of ewes that lambed in two consecutive cycles.

### 2.4 Statistical analysis

Data were statistically analysed using SAS (Version 9.2, SAS Institute Inc. Cary, NC 27,513, USA) software. Data were analysed by Kolmogorov–Smirnov test for evaluating the normality of distribution. The P4 concentrations during study were analysed based on repeated measurements by mixed procedure of SAS software. Comparisons of P4 concentrations in identical sampling times, weight of lambs and litter size among different groups were performed by one‐way ANOVA and LSD post hoc test. A few animal samples were excluded from analysis, because their serum P4 concentrations were outlier (an interval spanning over the mean plus two standard deviations). The percentage of the ewes with serum P4 > 1 ng/ml on day 0 (at injection of the first dose of PGF2α), conception rate, lambing rate, foetal mortality and twining rate of treatment and control groups were statistically compared using the Chi‐square test. If the 2 × 2 table had at least one expected cell count less than 5, then the Fisher exact test was used. The ovulation rate was determined based on the P4 concentrations of animals on day 17 (seven days after injecting the second dose of PGF2α), and was compared among studied groups using the Chi‐square test. Data were presented as the percentage or mean (±SE), and values of *p* ≤ .05 were considered as significant data.

## 3 RESULTS

### 3.1 Progesterone concentrations

**FIGURE 2:**
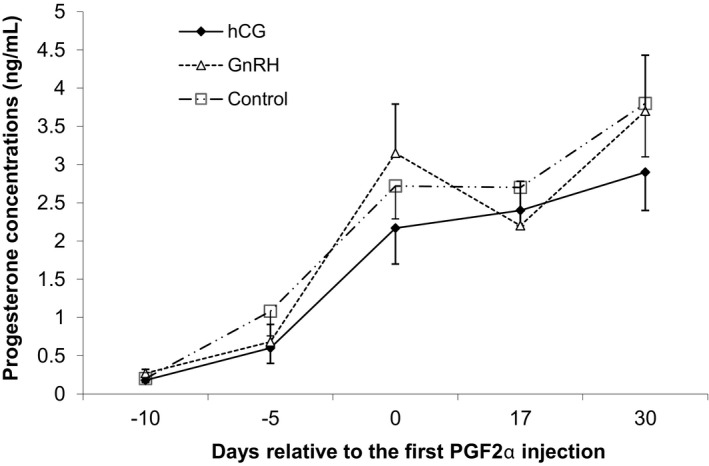
Serum progesterone (Mean ± SE) concentrations of ewes treated with prostaglandin and GnRH or hCG combined with the male effect in seasonal anestrous ewes

The results of P4 concentrations analysis based on repeated measurements are shown in Table 1. The time effect on the increasing of progesterone changes was significant during the study (*p* < .05). The mean of P4 concentrations of different studied groups is shown in Figure 2. Analysis of P4 concentrations between days ‐ 5 and 0 relative to the first PGF2α dose showed no significant differences among treatment and control groups (*p* > .05; Figure 2). There was no significant difference among groups in serum P4 concentration on days 17 and 30 after the first dose of PGF2α injection (Figure 2; *p* > .05).

**TABLE 1:** Effect of variables on the P4 concentrations (repeated measurements) during the study in seasonal anoestrous ewes

Effect	NumDF	DenDF	Fvalue	*p*value
Time	4	259	27.84	<.0001
Group	2	62.6	1.23	.3
Time*Group	8	259	0.46	.9
BCS	2	61.5	1.15	.3
Age	3	61.1	0.57	.6

Percentage (number) of ewes with serum P4 > 1 ng/ml on day 0 (first dose of PGF2α injection) was 71.4 (20/28), 67.9 (19/28) and 65.4% (17/26) in hCG, GnRH and control groups, respectively (*p* > .05). There was no significant difference in ovulation rate of studied ewes based on the P4 concentrations of animals on day 17 (*p* > .05; Table 2).

**TABLE 2:** Reproductive performance in first and second oestrous cycle of ewes treated with prostaglandin and GnRH or hCG combined with the male effect in seasonal anoestrous ewes

	hCG	GnRH	Control	*p*value
Ovulation rate % (*n*)	89.3 (25/28)	72.4 (21/29)	80.8 (21/26)	.3
Conception rate % (*n*)^a^	93.8(30/32)	90(27/30)	87.1(27/31)	.6
Lambing rate % (*n*)^b^				
The first cycle % (*n*)^c^	78.1 (25/32)	73.3 (22/30)	58.1 (18/31)	.2
The second cycle % (*n*)^d^	9.4 (3/32)	13.3 (4/30)	22.6 (7/31)	.3
Overall % (*n*)	87.5 (28/32)	86.7 (26/30)	80.6 (25/31)	.7
Twining rate % (*n*)	10.7 (3/28)	11.5 (3/26)	16 (4/25)	.8
Fetal mortality rate % (*n*)^e^	6.7 (2/30)	3.7 (1/27)	7.4 (2/27)	.8

^a^ Conception rate = (number of pregnant ewes at pregnancy diagnosis/number of exposed ewes) × 100

^b^ Lambing rate = (number of ewes lambing/number of exposed ewes) × 100.

^c^ Ewes lambed from 151 to 159 days after ram release.

^d^ Ewes lambed from 166 to 181 days after ram release.

^e^ Fetal mortality rate = [(number of detected embryos at pregnancy diagnosis—number of lambs born)/number of detected embryos at pregnancy diagnosis] ×100.

### 3.2 Reproductive performance of ewes

Table 2 shows the reproductive indices in the studied ewes. Lambing rate at the first cycle (induced oestrus after PGF2α treatment) tended to increase in hCG (78.1%) group compared with control group (58.1%; *p* < .1; Table 2). No significant differences in the other reproductive indices (Ovulation, conception and foetal mortality rate) were found among the studied groups (*p* > .05; Table 2). The number of lambs born per lambed ewe was similar among groups (*p* > .05; Table 3). No significant differences in birth weight of lambs were found among groups (*p* > .05; Table 3).

**TABLE 3:** Litter size in first and second oestrous cycle and weight of lambs (Mean ± SE) of ewes treated with prostaglandin and GnRH or hCG combined with the male effect in seasonal anoestrous ewes

	hCG	GnRH	Control	*p* value
Litter size^a^				
The first cycle (*n*)^b^	1.08 ± 0.05	1.14 ± 0.07	1.16 ± 0.09	.7
The second cycle (*n*)^c^	1.33 ± 0.33	1.0 ± 0.0	1.14 ± 0.14	.5
Overall (*n*)	1.11 ± 0.06	1.12 ± 0.06	1.16 ± 0.07	.8
Litter birth weight				
Birth type				
Singleton (kg)	4.77 ± 0.13	4.56 ± 0.12	4.82 ± 0.10	.2
Twin (kg)	4.93 ± 0.18	5.33 ± 0.46	5.15 ± 0.40	.7
Lamb sex				
Female (kg)	4.58 ± 0.15	4.38 ± 0.14	4.73 ± 0.13	.2
Male (kg)	5.03 ± 0.16	4.91 ± 0.18	5.06 ± 0.11	.8

^a^ Total number of lambs/total number of ewes lambing in each group (Lambs/lambed ewe).

^b^ Lambs/lambed ewe that conceived at induced oestrus.

^c^ Lambs/lambed ewe that conceived at first spontaneous oestrus.

## 4 DISCUSSION

The results of the present study showed that the lambing rate at the first cycle tended to increase in ewes treated with prostaglandin and hCG in combination with the ram effect when compared with control ewes during the out of breeding season. In this study, GnRH and hCG treatments did not improve ovulation in seasonal anestrus ewes. Although Gonzalez Alvarez et al. (2016) stated that gonadotrophins are required to support the growth of follicles. Husein, Ababneh, and Haddad (2005) reported that an injection of GnRH may increase LH, which in turn may induce ovulation or follicular atresia (Husein et al., 2005); current results indicated that follicular regression has occurred, due to similar ovulation rate in all groups. These results are in agreement with González‐Álvarez et al. (2016), which stated that large follicles may suffer regression when applied GnRH or hCG in a PGF2α‐based protocol. Similarly, Almadaly, Ashour, El‐Kon, Heleil, and Fattouh (2016) found no positive effect on ovulation synchronization of ewe with GnRH ‐ PGF2α ‐ GnRH during the out of breeding season in ewes (Almadaly et al., 2016). So, the effect of exogenous GnRH injection may vary depending on the stage of the oestrous cycle of treated animals (González‐Álvarez et al., 2016).

In this study, we used a single dose of hCG during non‐breeding season in non‐synchronized ewes. Therefore, it could not change the P4 concentrations of treated ewes, and tended to increase in lambing rate and tended to decrease in lambing period was found compared with other groups. Three administrations of hCG in eCG superovulated and P4‐synchronized ewes improved CL characteristics and increased the total number of CL and serum P4 concentrations during the breeding season (Shabankareh, Seyedhashemi, Torki, Kelidari, & Abdolmohammadi, 2012). Fernandez et al. (2019) reported that the injection of GnRH on day four of post artificial insemination (AI) improved litter size and weight; however, it did not improve pregnancy rate. The injection of GnRH or hCG on day four of post AI induced and increased the formation of accessory corpora lutea and serum P4 concentrations in treated ewes during the breeding season (Fernandez et al., 2018). It seems that the consideration of dose and time of hCG administration are important for improving the luteal activity. The dose‐ and time‐dependent effect of hCG on luteal function was reported in anoestrous ewe lambs by Catalano et al. (2012).

In this study, in agreement with Kaya, Kaçar, Kaya, and Aslan (2013), the injection of hCG and GnRH had no effect on the lamb weights. It was also observed that the hCG and GnRH injection did not affect the litter size during the anoestrous period. The results of the total lambing rate of treated ewes obtained in this study were similar to those reported by Mirzaei et al. (2017), who examined the combined use of a single dose of GnRH and PGF2α administered together with the ram effect during non‐breeding seasons. Similar to previous studies (Kaya et al., 2013; Saharrea et al., 1998), we also found that the GnRH had a lower effect on fertility than hCG in treated animals.

González‐Álvarez et al. (2016) suggested that the administration of hCG and PGF2α in progesterone primed goats was the best option to induce and synchronize estrus as well as ovulation during the anoestrous season. Dursun (2019) reported that the injection of GnRH or hCG in synchronized ewes (not pregnant after multiple matings) with sponge combined with pregnant mare serum gonadotropin and PGF2α increased the profitability of flocks at the end of the breeding season. During the breeding season, a double administration of GnRH (at device insertion and 56 hr after CIDR removal) instead of eCG had similar P4 concentrations and fertility rate in a protocols based on short‐term (five days) CIDR treatment (Martinez‐Ros & Gonzalez‐Bulnes, 2019). Improved oestrous synchronization and fecundity were reported in ewes receiving GnRH–PGF2α protocol when progestagen sponge was applied during treatment at the beginning of the breeding season (Titi, Kridli, & Alnimer, 2010). Husein and Kridli (2003) reported that the progesterone priming prior to a GnRH‐PGF2α treatment of anoestrous ewes may enhance follicular growth and increases their response in oestrus and pregnancy rates. It can be concluded that the effect of GnRH or hCG administrations on the luteal activity and fertility depends on the ovarian activities of treated ewes (cyclic or anoestrous ewes). It is important that the progesterone priming may be responsible for the improved response of treated ewes with hCG and GnRH.

Pregnancy loss was reduced in synchronized low‐prolific Rahmani ewes using a double injection of PGF2α by treatment of GnRH at the time of estrus or 7 days post‐mating (Hashem, El‐Azrak, et al., 2015). Tighter synchrony of ovulation was reported in treated ewe with ovsynch‐protocol compared with double PGF2α injection; although, conception rate and litter size did not differ between the two regimes (Hashem, El‐Zarkouny, Taha, & Abo‐Elezz, 2015). In this study, similar to study of Alnimer, Tabbaa, Amasheh, and Alzyoud (2005), there was no difference in conception and the first lambing rates between hormonal treated ewes and the ram effect. Alnimer et al. (2005) reported that the ewes may be synchronized using either GnRH‐PGF2α program or two injections of PGF2α 10 days apart. Ovarian activity of postpartum (35 to 60 days after lambing) ewes was resumed by the ram effect (Ferreira‐Silva et al., 2017). So, the male effect is efficient to induce ovarian activity and increase progesterone concentration in the control ewes.

In conclusion, the administration of prostaglandin and hCG in combination with the ram effect tended to decrease lambing period. In other words, this protocol tended to increase lambing rate at the first cycle. Treatment with hCG or GnRH did not increase serum P4 concentrations of treated Karakul ewes during the non‐breeding season.

## 5 ETHICAL APPROVAL DETAILS

This experiment was approved by the Ethical and Research Committee of the School of the Veterinary Medicine, Shiraz University (96GCU3M83440).

## CONFLICTS OF INTEREST

The authors declare no conflicts of interest and financial, personal or other relationships with other people or organizations.

## AUTHOR CONTRIBUTION

**Mohammad Ayaseh:** Data curation; Investigation; Writing‐original draft. **Abdolah Mirzaei:** Formal analysis; Methodology; Project administration; Supervision; Validation; Writing‐review & editing. **Alidad Boostani:** Data curation; Investigation; Resources. **Mansur Mehrvarz:** Data curation; Investigation.

## AUTHOR CONTRIBUTIONS

Mohammad Ayaseh is responsible for clinical examiner, data sorting and wrote the manuscript. Abdolah Mirzaei designed and performed the study, analyzed the data and wrote the manuscript and revision. Alidad Boostani is coordinator for all part of this project. Mansur Mehrvarz helps as assistance for clinical examination and data collection.

### PEER REVIEW

The peer review history for this article is available at https://publons.com/publon/10.1002/vms3.353.

## Data Availability

The data that support the findings of this study are available from the corresponding author upon reasonable request.
